# Plague, Religion and Urban Space in Sixteenth-Century Antwerp

**DOI:** 10.1093/shm/hkad090

**Published:** 2024-03-14

**Authors:** Janna Coomans, Léa Hermenault, Rogier van Kooten, Claire Weeda

**Affiliations:** Department of History, Utrecht University, Utrecht, The Netherlands; Department of History, Center for Urban History, University of Antwerp, Antwerpen, Belgium; Department of History, Center for Urban History, University of Antwerp, Antwerpen, Belgium; Department of History, Leiden University, Leiden, The Netherlands

**Keywords:** plague, public health, religion, Low Countries, GIS

## Abstract

Antwerp’s response to the outbreak of plague in the 1570s offers new insights into the effects of epidemics on urban communities in relation to their religious, economic, and spatial fabric. Antwerp’s transition from a Catholic to Calvinist government in 1577, and back to Catholicism in 1585, allows us to study its reaction to and the effects of plague across religious boundaries within a short time span. Using GIS, we have compared various rich datasets concerning plague: the register of houses locked in quarantine; the health certificates issued by authorities; plague fatalities recorded in St. Jacob’s parish; a wide range of urban regulations; and information about the size of households, their composition, rents and real estate values in Antwerp. Combined analysis shows that Catholics and Protestants, whose houses were concentrated in different city districts and who had distinct professional and economic profiles, experienced plague quite differently, both physically and spiritually.

In the turbulent 1570s, the metropolis of Antwerp was caught in the midst of a religious and civil war and a revolt against the Spanish Habsburg rule of the Low Countries. During the 1576 Sack of Antwerp (also known as the ‘Spanish Fury’), disgruntled Spanish soldiers awaiting payment massacred thousands of citizens and vandalised the city. The violence was considered a watershed moment in the Eighty Years’ War (1568–1648), and in its aftermath the rebellious provinces in the Netherlands unified and signed the Pacification of Ghent treaty with the aim of removing all the Spanish soldiers from their territories.^1^ Between 1576 and 1584, Antwerp subsequently became a stronghold of the Dutch States faction and in December 1577 shifted allegiance from a Catholic to a Calvinist government. After a short-lived attempt to install ‘freedom of religion’ in 1578, the city forbade the public practice of Catholicism in July 1581—the apex of the growing popularity of Calvinism and Lutheranism in Antwerp in the previous decades.^2^ This situation lasted until 1585, when Antwerp’s magistrates once again embraced Catholicism. Amidst this turmoil, however, another calamity unfolded that has gone almost unmentioned in the history books: a string of outbreaks of plague.^3^ Antwerp’s response to the plague epidemic of the 1570s offers new insights into how a city’s religious, economic, and spatial fabric affected its reaction to the disease. The city’s transition from a Catholic to a Calvinist government in 1577, and back to Catholicism in 1585, makes it possible to study its responses to and effects of plague across religious boundaries within a short time span.

Reactions to the outbreak of plague were informed by different religious perspectives and spatial and social-economic factors. To understand how these various perspectives and factors interacted, this article looks at the public health interventions Antwerp’s authorities imposed upon its population, such as the regulation of processions, the issuing of health passes and the locking up of infected houses, while transitioning from Catholicism to Calvinism. This information has been overlaid across the city’s topography and its religious and socio-economic configuration. We argue that plague’s footprint was shaped significantly by socio-economically generated factors—different living conditions in different districts—that corresponded with the clustering of different religious groups.^4^ Districts where Catholics made up the majority of the population felt the effects of restrictions on movement more acutely. During the transition to a Calvinist government between 1577 and 1585, the cessation of plague processions and the limited access to plague shrines also constricted the Catholic population’s spiritual strategies to deal with disease.

The outbreak of plague in Antwerp (caused by the bacteria *Yersinia pestis*) in the 1570s was quite severe.^5^ In the summer of 1571, the Lutheran legal scholar Jan van Wesenbeke commented in his chronicle, which runs from 1567 to 1580: ‘In August, September, October and November in the year 1571, there was such a high number of fatalities from plague here in the city and its surroundings, that was not seen in sixty years, or even in our memory’.^6^ The painter and Lutheran supporter Godevaert van Haecht similarly claimed that 1700 people died from plague in Antwerp in September 1571. The casualties were mostly children, who had also been brought to the hospital from outside the city.^7^ If correct, Godevaert’s estimate means that 1.5 to 2 per cent of the population died from plague in that year, based upon the 1568 population count of 90,000–104,000.^8^ In the early decades of the twentieth century, A.F.C. van Schevensteen, a historian and physician appointed at the Ooglijdersgesticht (ophthalmic hospital) in Antwerp, echoed these observations. He recorded that in the summer of 1571, the plague, first erupting in Turnhout, spread ‘like wildfire’. It was precipitated by a flurry of ordinances issued by the Antwerp authorities. In response, an extra cemetery was founded in Nieuwstad, the city’s new district on the northern boundary. The city organised processions and a confraternity dedicated to St. Roch (St. Rochus in Dutch) was established in the Cathedral of Our Lady (Onze-Lieve-Vrouw Kerk).^9^ Plague’s presence in the city was thus manifest, although in this instance the mortality rate was much lower than compared to the major outbreaks of plague during the fourteenth century in other regions in Europe.^10^ The disease resurfaced in 1574 and, with ferocity, in 1578. On 12 May 1582, the graveyard of St. Andries was so overrun with the bodies of plague victims buried in haste, stacked on top of one another and sometimes ‘shamefully’ exposed, that the mounds of soil allegedly were heaped up above the level of the walls. The stench was said to have led to the outbreak of various diseases in the same quarter (*diversche contagieuse siecten*), and was so severe that the inhabitants asked for a new graveyard.^11^ In 1583, there were extensive deliberations among the city magistrates and almoners about the burial of corpses in the garden of the Falcon monastery (Falcon klooster) in Nieuwstad, where earlier in 1580 the establishment of a new plague hospital was ordained.^12^

At the time, about half of Antwerp’s population was Protestant, of whom in 1585 in a survey of the city’s militia about two-thirds were Calvinist and one-third Lutheran.^13^ As merchants and traders, the Protestants’ dwellings were concentrated mostly in the city centre near to the old exchange market (Old Bourse) and the main market square (Grote Markt; see Figure 1).^14^ According to Alfons Thijs, from the 1550s Antwerp’s Catholic magistrates had exercised a policy of appeasement of the Calvinists and Lutherans in the city, because of their economic significance as foreign merchants, traders and craftsmen.^15^

**Fig. 1.**
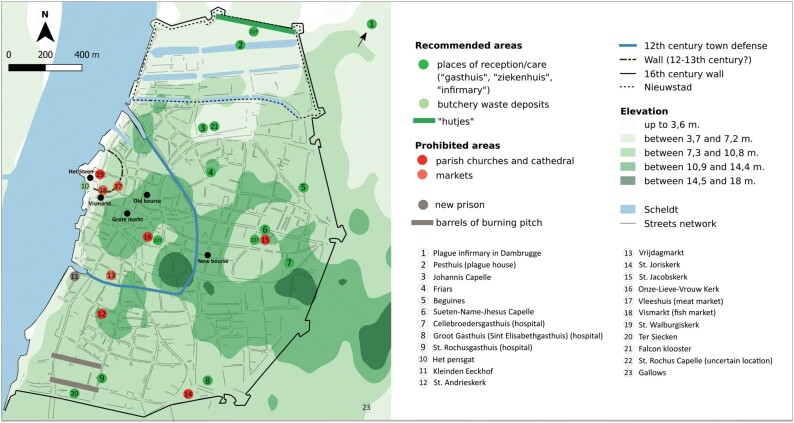
Antwerp’s general topography and plague institutions

To date, historians have paid relatively little attention to how religious differences within Christendom influenced how people dealt with plague and its impact. Ronald Rittgers studied the outbreak of plague in Nuremberg in 1562–3, a city that was an important stronghold of the Lutheran confession since 1525. Rittgers argues that the city’s response to plague was in many ways traditional, continuing to decree that citizens avoid large crowds, corrupt matter or stench. The governors advised to apply the so-called non-naturals, external agents such as fresh air, a balanced diet, exercise and rest.^16^ These were interventions that had been developed based on the medical ideas of the Graeco-Roman physician Galen (129–199).^17^ The main, new distinction was the rejection of the belief in the intervention of the saints, such as Sebastian and Roch. The city forbade singing the Salve Regina or holding processions and private masses to ward off plague.^18^ Protestants did not consider suffering to be a good work but rather a test of faith, placing themselves directly in God’s hands on the basis of faith alone and through Christ alone. As Rittgers observes, in practice, saints accordingly disappeared from the letters of citizens after 1525, as did cult objects from their households. Yet this certainly did not mean a rejection of medical interventions such as the imposition of quarantine.^19^ Nonetheless, as Philip Rieder comments, Geneva’s transition to Protestantism in 1536, and the ensuing influx of high numbers of foreign Protestant merchants, physicians, surgeons and apothecaries—similar to Antwerp—reconfigured its medical landscape, leading to a ban on traditional and magical healing practices. Many inhabitants in Geneva subsequently took to walking to nearby Catholic sanctuaries outside the city to visit ‘traditional’ healers.^20^

Attempts to contain plague have been studied extensively, particularly for Italian Catholic cities rich in sources. A large body of scholarship has explored the nature of the prophylactic sanitary and spiritual measures taken to reduce its spread.^21^ Across Europe, city governments sought to separate the sick from the healthy by means of quarantine and to reduce disease transmission by limiting the mobility of people and goods. In particular, they strove to reduce exposure to disease-bearing miasma (poisonous vapours coming from corrupt matter and thought to spread the disease) by taking strict hygiene measures that increasingly became related to social conditions. Ann Carmichael argues that in the course of the fifteenth century in Florence, the conviction grew that there was a link between poverty and plague. John Henderson’s *Florence under Siege* shows how this city organised extensive sanitary surveys surrounding the 1629–31 outbreak of plague, turning its gaze to the housing conditions of the city’s poor.^22^ Beyond the relationship between poverty and plague, Samuel K. Cohn Jr.'s *Cultures of Plague*, focusing on the impact of plague in Italy in 1575–8—coterminous with the outbreak in Antwerp—observed how the outbreak moved health boards to track its spread, a practice for which we have no evidence for Antwerp. In Italy, Cohn argues, Counter Reformation Catholicism reinforced civic responses.^23^ These studies have been enriched with research on the establishment of plague hospitals and substantial interdisciplinary studies of plague’s pathologies and mortality rates.^24^ However, beyond the paucity of studies on religious transformation and plague, there is also a significant lacuna in our understanding of responses to plague in the highly urbanised region of the Low Countries.^25^ This is reflected in the fact that, despite its severity and frequent recurrence, only a handful of articles about plague in Antwerp have appeared in print.^26^

This silence is all the more remarkable given that Van Schevensteen published two volumes, in 1931–2, presenting a wealth of sources about plague in Antwerp between 1454 and 1792.^27^ A striking cluster of documents survives for the years 1570–80, revealing the city’s multi-layered handling of the epidemic. Firstly, the parochial burial register of the St. Jacobskerk (St. James) in Antwerp, dating to 1568–80, contains records of the parish burials between 1569 and 1580, with a separate registration of plague deaths between 1570 and 1577, often mentioning, in addition to the date of death, the name, location and profession of the deceased.^28^ Secondly, the register of certificates lists the men and women who requested health passes in 1571–2, detailing their names, professions and the sites they visited.^29^ The health passes allowed applicants to move freely in and out of the city, despite the restrictions on movement that had been imposed by the authorities. Thirdly, one of the city locksmith’s registers has survived, revealing which households were considered to be infected with plague and locked between 1579 and 1580.^30^ Fourthly, a large body of ordinances and financial accounts sheds light on the city’s plague policies during the transition from Catholicism to Calvinism. Finally, chronicles offer a granular view of Antwerp’s diverse population—young, old, female and male, wealthy and poor, citizen and migrant, Catholic, Calvinist and Lutheran—who experienced plague differently. The chronicles shed light on the social-religious tensions and inequalities that the outbreak of plague enlarged.

Antwerp also offers quite an exceptional case study for the Low Countries mainly because the data on plague can be integrated with relatively precise (cadastral) information concerning the names and locations of houses and the value of real estate.^31^ These datasets have been processed using a Geographical Information System (GIS).^32^ Antwerp’s sources add an extra layer of complexity because the detailed data available concerning the city’s religious-spatial and socio-economic stratification can be combined with information about the size of infected houses and locations of plague fatalities. This gives insight into how different communities were exposed to plague differently, across the religious and spatial fabric of the city, running along socio-economic fracture lines.

## Regulating Plague in Antwerp’s Cityscape

But before turning to the response to plague in the 1570s, it is helpful to first outline Antwerp’s cityscape and the evolution of its plague policies. Situated on the Scheldt on the border between Brabant and Flanders, Antwerp was from its early days a gateway to the North Sea. Consequently, the city developed as a trading hub during the later Middle Ages. In the sixteenth century, population numbers surged to circa 100,000. Yet Antwerp’s spatial layout continued to be determined by its growth in the preceding centuries (see Figure 1). From the ninth century, Antwerp emerged around a castle (Het Steen) and a semi-circular fortification close to the river. A *vicus* developed on the southern bank, and kept extending to the south and the west until the 1540s–50s, when Holy Roman Emperor Charles V built new fortifications. Accordingly, Antwerp’s key religious and political sites: the Cathedral, the town hall and the Old Bourse, were all situated within the twelfth-century fortifications.^33^

**Fig. 2.**
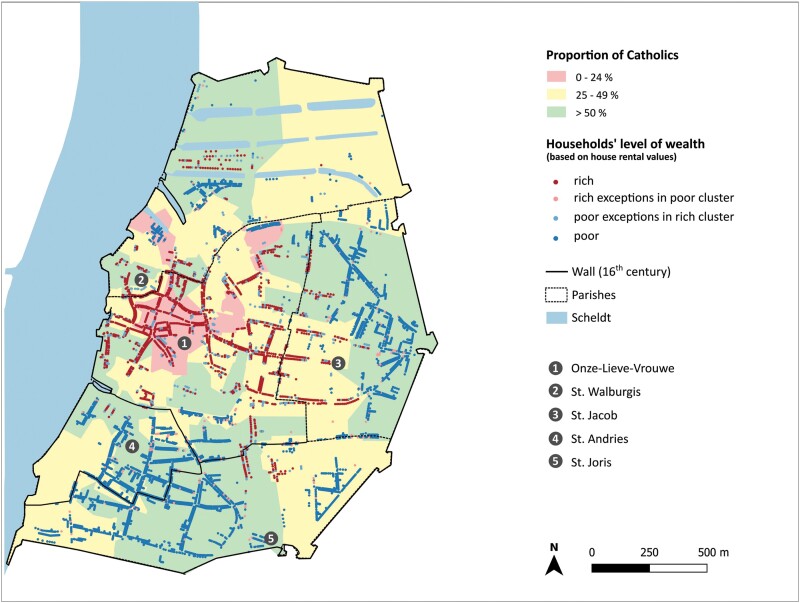
Catholicism and Protestantism in Antwerp per quarter^36^

Although in the sixteenth century, the various religious groups by no means lived strictly segregated, Antwerp’s neighbourhoods do show a clear pattern of concentration of Protestant and Catholic residents, which suggests a correlation between religious and social stratification (Figure 2).^34^ Generally speaking, the central neighbourhoods of the first district housed the largest clusters of Protestant households, while the very poor districts in the south and east of the city were dominated by Catholics. There was a significant Protestant ‘enclave’ around the ‘Nieuwe Waag’, where merchant goods were weighed. This is a typical example of early gentrification, propelled by the building projects of Gilbert Van Schoonbeke and the high number of wealthy Protestant merchants in this neighbourhood.^35^

**Fig. 3.**
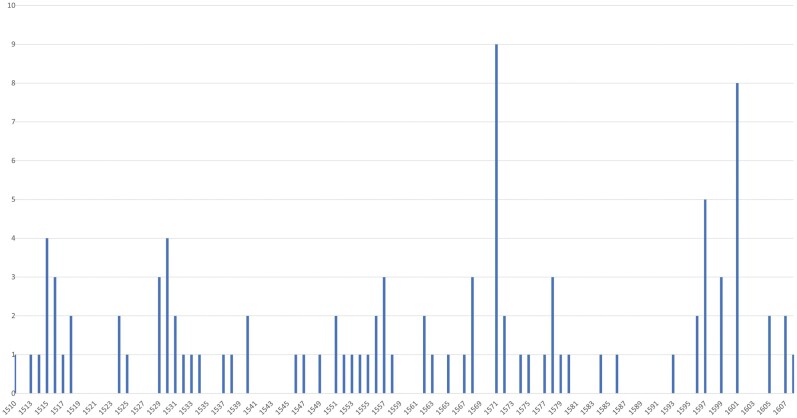
Number of plague ordinances issued per year

The urban fabric was micromanaged by a magistracy that functioned semi-independently from its seignorial overlords (the Dukes of Brabant and later Habsburg Emperors), with its own courts and laws. Between 1489 and 1610, Antwerp’s magistrates issued more than 3,800 ordinances in total, pertaining to a broad range of topics, which are documented in the *gebodboeken*.^37^ In the sixteenth century, a considerable number of these ordinances dealt with the physical and moral health of the city.^38^ Some clusters of bylaws suggest a heightened presence of plague (called *pestilentie* or *haestige siekte*) within the city at certain moments: in 1515 (4 ordinances), 1516 (3), 1530 (4), 1571 (9), 1597 (5) and 1601 (8).^39^ In total, on over one hundred occasions (Figure 3), the magistrates issued regulations attempting to halt the further spread of the plague within the city, or to avoid its influx from other cities and the countryside. These regulations echo those of Dubrovnik and northern Italian cities, such as Milan or Pistoia, despite the absence of health boards in Antwerp at this time. This is perhaps less surprising if we bear in mind that Antwerp maintained intense contacts with an international trade community, and that there was a strong presence of Italian merchants in the city.^40^ Moreover, physicians from the Low Countries and Italy often exchanged medical ideas in the late sixteenth century and many scholars from the Low Countries studied medicine in Italy.^41^

How did the transition from a Catholic to a Calvinist government in 1577 influence plague regulations? The clearest point of comparison are the decrees issued in 1571 (40 measures issued at once) and in 1578 (34 measures, shortly after the Calvinists took over government). On aggregate, if we compare the latter ordinance to previous regulations, relatively little changed. In fact, the vast majority of sanitary measures issued in 1578 were copied verbatim from those of 1571. Thus, just as Ruth MacKay argued that life went on during the devastating Castilian Plague of 1596–1601, so too did the city government in Antwerp make only minor adjustments to its regulations of hygiene and containment when it transitioned from Catholicism to Calvinism.^42^ Echoing the developments in Geneva documented by Philip Rieder (mentioned above), Antwerp’s city government did issue an additional call to supervise medical practitioners, however, claiming their incompetence ‘strongly increases the spread (*grootelyck verbreyt*) of disease’.^43^ In 1578, the 1571-ban on weddings, banquets or fairs attended by more than twelve people was annulled, yet the injunction forbidding parents to take their children to school during an outbreak remained in force.^44^ The sections on sanitation and food markets (including slaughtering) were also copied verbatim.^45^

Like so many urban governments and medical authorities across Europe, Antwerp’s magistrates continued to regard the corruption of air, recognised by its stench and the presence of vermin, as one of the main causes of disease. Miasma came from corrupt matter or poisonous vapours shed by the movement of heavenly bodies, ultimately driven by divine wrath. Sanitary efforts were, therefore, in part geared towards reducing dirt and stench.^46^ Once the plague had arrived, sanitary measures aimed to contain its spread.^47^ The disease was also considered to spread person-to-person and via contaminated goods, notably textiles and fruit. Both people and objects ‘corrupted’ their immediate surroundings. The magistracy accordingly acted to restrict the mobility of and interactions between those infected with plague and the city’s population at large. Rather than a sign of ineffectiveness, the repetitious nature of Antwerp’s plague regulations meant they created continuity and the ability to respond quickly to outbreaks. Yet difficulties with enforcement did seem to occur. Plague rules ‘had not been fully observed’, the magistrates lamented both in 1571 and 1578, although this might be a cliché employed by the city government.^48^ More concretely, in 1571, the city issued a warning not to assault the *cellebroeders*, the religious confraternity (also called Alexians) who, alongside their female counterparts, the *Swarte Susters*, were responsible for caring for and transporting dead plague victims at night at the behest of the city authorities.^49^

Were any changes implemented during the transition to Calvinism concerning plague facilities or institutions of care? Plague facilities in Antwerp had existed since at least the late fifteenth century (see Figure 1). In 1487, the city had ordained ‘that from henceforth, no plague sick shall be laid on the city fortifications and that those lying there should be moved’.^50^ The St. Roch hospital (St. Rochusgasthuis) was in operation from at least 1496; in 1532 its rooms were expanded. The sick were admitted by consent (and in exchange for a fee) by the *aalmoezeniers* (almoners), with a letter in hand from officials known as the *keurmeesters*.^51^ We also know that a confraternal house of the *cellebroeders* was situated, from 1527, in the Vlemincks-straete.^52^ Upon the request of the local inhabitants—who were perhaps fearful of contamination—their house was later moved to the Lange Nieuwstraat, where they settled in 1548. In 1549, a plague hospital was built in the Nieuwstad at the Slijck Poort.^53^

Both Catholic and Calvinist magistrates were concerned about the lack of health care spaces and facilities for (poor) plague patients. Beyond the plague hospital set up in the Nieuwstad in 1549, two health care institutions took on the main burden of caring for plague patients: the St. Roch’s hospital and St. Elisabeth’s hospital (St. Elisabethgasthuis). In 1512, a separate space (*pestilencyhuys)* for plague victims had been created at the latter. However, these cordoned off spaces tended to lose their distinct character when the number of patients surged during times of crisis—epidemics, but also military clashes generated a major influx of sick and wounded inmates. The overcrowding of St. Elisabeth’s hospital had led to its reputation as being a hotbed of plague. On several occasions in the mid-sixteenth century, the hospital was accused of mismanagement. Around 1580, when the Calvinists seized power, they devised a plan to build a ‘new house’ for plague victims in the Nieuwstad, and a year later discussed the possibility of moving plague patients to the *Predikherenklooster.* However, neither of the projects was fully realised. Likewise, initiatives to enlarge the capacity of St. Elisabeth’s hospital, which had about a hundred beds but often several hundred inmates, proved difficult to put into practice.^54^ In 1581, the St. Roch hospital was also seized by the Calvinist government. Plague sufferers were still treated at the hospital, which now fell under the auspices of a ‘concierge’.^55^

Further traces of changes made in the course of the religious transition are found in a letter of municipal consent from July 1586, a year after the city reverted back to Catholicism. In previous years, the clerics of the Ter Zieken cloister had occupied the St. Roch hospital, suggesting the latter was no longer, or at least not fully, in use as a plague hospital. In 1586, the two remaining clerics relocated to their original dwellings, which had been partly destroyed. The St. Roch hospital was once again vacant, which was important ‘since plague had been detected in the orphanage and hospital for the mentally ill (*dulhuys*)’.^56^

The multiple changes in Antwerp’s plague hospitals fit into a broader picture characterised by variety. While plague hospitals were increasingly established throughout Europe, parallel to the more trade-oriented quarantine stations of the Italian *lazaretti*, they often initially were temporary, makeshift or otherwise ad hoc initiatives, put into place during epidemics but changing in function when instances of plague infections diminished. The distinct architecture and situation of plague facilities *outside* the city was more a seventeenth-century innovation, and rare before that period, at least in the Low Countries.^57^

Most offices dealing with plague remained the same across the Catholic/Calvinist transition, although we do not know whether they were held by different people. In the fifteenth century in Italian cities like Milan, health boards were installed to manage and advise on public health issues.^58^ In Antwerp, rather than health boards, so-called *keurmeesters* were the main officials in charge of enforcing *all* of the city’s ordinances, including plague regulations, and these men had the authority to prosecute offenders.^59^ The *keurmeesters* decided which houses were designated as plague-infected and coordinated the closing and supervision of them.^60^ Across the period of religious transition, the *keurmeesters* retained these competences.^61^

Alongside the *cellebroeders*, a standard entry in Antwerp’s city financial accounts are the plague physician (or surgeon), plague midwife, and the *pestmeester.* The latter administered care and carried out various policing tasks, as a kind of assistant to the *keurmeester*.^62^ These officials were responsible for detecting and diagnosing plague in households and transporting patients, and the deceased, out of their houses (the movement of the bodies, an ordinance detailed, needed to happen at night).^63^ They also cared for the plague sick.^64^ In this context, following the temporary switch from a Catholic to a Calvinist government in 1577, the city did however implement a striking change concerning the appointment of plague officials. In 1583, four ministers (*parochianen*) were hired to ‘console the plague sick [...] without making any distinction’ (*sonder onderscheyt*), and to visit all religious and charitable houses and hospitals.^65^ This provision of spiritual care seems to have replaced older Catholic initiatives, and coincides with the temporary moratorium on processions under the Calvinist government, discussed below.

Since 1555, in times of plague, the city also appointed a locksmith ‘to close and reopen houses’. Thus, in 1575, Jacob Jansse was paid 50 lb for locking and unlocking ‘infected houses’.^66^ In 1580, Antwerp rewarded Lambrecht Platvoet for the same services rendered between 1579 and 1580. Fortuitously, the list of addresses of houses sealed off by Lambrecht has survived, falling within the transition period from Catholicism to Calvinism. Nonetheless, this specific register was probably one in a series beginning in 1555 that survived by coincidence or as a result of more cautious archival conservation practices under the Protestant regime.^67^ From 1580, so-called *toesienders* also appear in the records. These individuals patrolled the locked houses and gave inhabitants locked inside food and drink. The office continued to exist after 1585, when the government switched back to Catholicism. For instance, in 1588, a man was paid for his services as a ‘*sluyter en toesiender*’ (for locking and patrolling).^68^

After 1599, a special servant of the *keurmeesters* (called the *knape der ghesontheyt*, servant of health) was hired as a plague inspector for a period of 6 months, but in later years, these servants turn up in annual lists of officials’ salaries.^69^ The servants of health oversaw the activities of the plague house cleaners, known as *schrobbers.*^70^ Their other tasks probably resembled those of servants or helpers (*knechten/gesellen*) of the *keurmeesters*, who sealed off infected houses with wooden bars or planks (*latten*), as they did in Italy.^71^

The authorities’ main focus in the regulations was maintaining the city’s economic vitality and the mobility of specific groups, notably merchants. The ordinances show little sensitivity to any perceived relationship between disease and poverty. In this, Antwerp arguably differed from Italian cities, whose physicians, Cohn argues, after the 1575–8 outbreak increasingly paid attention to social conditions.^72^ Thus, at first glance, Antwerp’s plague regulations suggest they first and foremost served the political and economic interests of the magistracy, rather than of the population at large.

## Spiritual Interventions

Antwerp’s authorities believed that miasma and contact between humans, goods and animals drove the spread of plague. Yet sin was considered to be the primary cause of it. Catholic magistrates accordingly considered spiritual care, appealing to the intercession of the saints, of paramount significance. Spiritual prophylactics meant the organisation of processions, confession and prayer.^73^ Through looking at and carrying in procession images and relics of saints such as St. Roch, believers solicited the protection of the heavens against the plague.^74^ By seeking spiritual alleviation, feeding oneself with the health-giving sacrament of the eucharist and cleansing the soul and passions, mortal beings hoped to strengthen their bodily stamina and keep debilitating, corruptive processes at bay. Prayer and processions represented atonement for the population’s sins, hopefully appeasing God’s wrath. As Marie-Louise Leonard argued, the linkages between the medical and the spiritual were omnipresent in civic measures taken in Italy.^75^ In Protestantism, however, believers were expected to place their faith directly in God’s hands.

As in other cities across Europe, the Catholic magistrates in Antwerp had taken to organising processions in the city as a form of ritual prophylactics from the turn of the sixteenth century. On 23 October 1518, Antwerp ordered a procession to be held to pray to God for the city’s deliverance from plague and other pests. It was repeated in 1524, and then held annually—not in direct response to actual outbreaks of plague but as an act of prevention—in the years 1529–33, 1536–8, 1540, 1546, 1551, 1558 and following years.^76^ In August 1571, a procession was held around the market square near the Cathedral, a district where many Calvinists dwelled. The participants carried the effigy of the Virgin Mary, with holy sacraments, praying to God to protect the city and surrounding villages. A large number of masses were sung with great devotion in God’s honour.^77^ However, from 1577, the processions in Antwerp ceased—as had happened in Nuremberg when that city became a Protestant stronghold. Directly after the Catholics regained power in 1585 in Antwerp, the processions were resumed.^78^ The moratorium on processions in the intermediate years robbed the Catholic population of an important prophylactic.

Before the transition to Calvinism, Antwerp’s population had access to shrines dedicated to plague saints. In 1512, the city had heaved a sigh of relief after an outbreak of plague seemingly was contained through the interventions of the local saint Fredegandus.^79^ However, it was the cult of St. Roch that was most actively developed in the city. In 1496, a hospital dedicated to him was founded under the auspices of the *aalmoezeniers*.

**Fig. 4.**
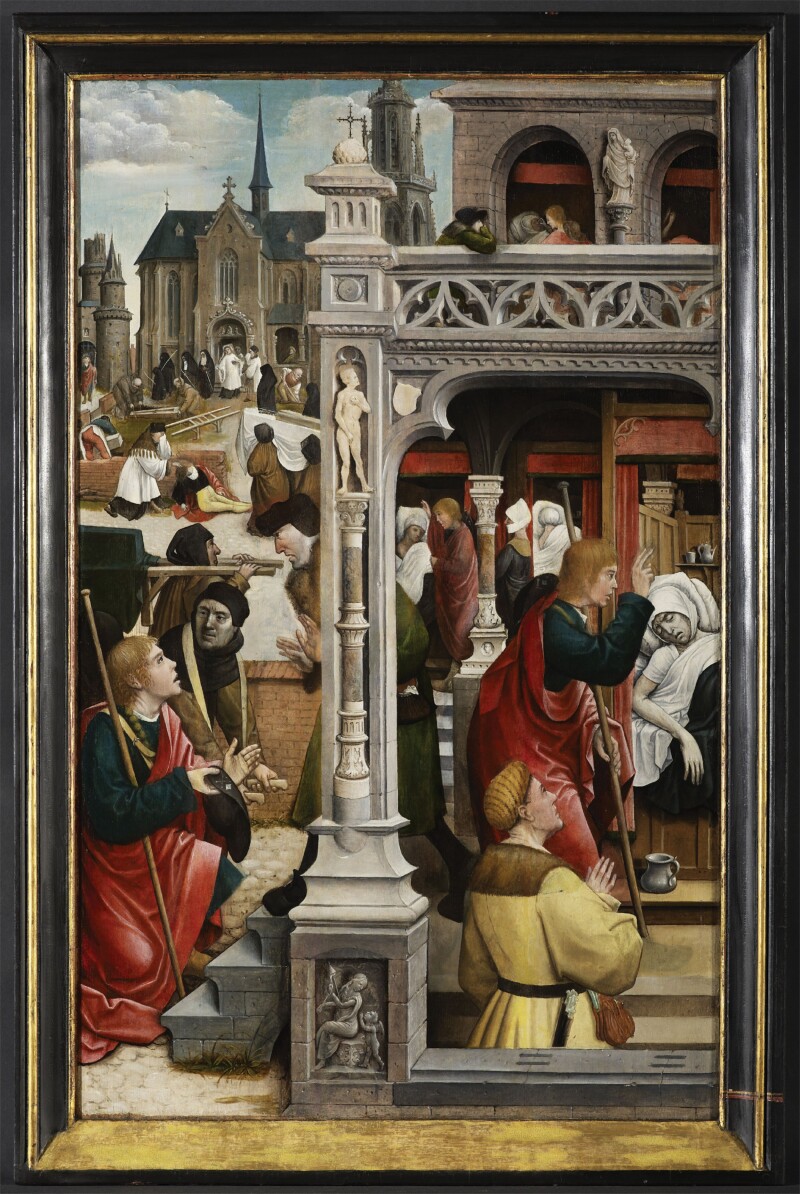
Detail of the retable in St. Jacob’s Church in Antwerp, 1517, showing plague sick and a plague burial. © KIK-IRPA, Brussels

The parish church of St. Jacob’s (St. Jacobskerk) also commissioned a chapel altar in the nave of its church dedicated to St. Roch. A spectacular altarpiece was placed at the altar presenting the life of the plague saint, of which twelve panels have survived (see Figure 4). Viewers reflected upon these images, recalling Roch’s suffering and seeking consolation in the healing powers of his relics. The shrine’s popularity, attracting lavish donations from those seeking protection against the disease, is evident from the conflict between the Guild dedicated to the Altar of St. Roch and St. Jacob’s churchwardens in the 1560s. The latter had in previous years attempted to seize part of the altar’s funds, in a period when donations to shrines and the sale of indulgences were in decline. The Guild devoted to St. Roch successfully petitioned King Philip II in 1563 to stop the syphoning off of the altar’s funds. Remarkably, the altar of St. Roch also was spared during the attacks on statues and images by iconoclasts, hired by Calvinist merchants, in 1566.^80^ The attackers desecrated all the other 18 altars in the St. Jacob’s Church. Calvinists ridiculed the procession in Antwerp bearing the effigy of Mary (Onze-Lieve-Vrouweommegang) and destroyed statues and images in the Cathedral, and in other churches, chapels and monasteries.^81^ In contrast, the altar of St. Roch was instead used in an attempt to reconcile Protestants and Catholics. On 13 September 1566, William of Orange (1533–84), political leader of the Dutch Revolt, attended mass at the altar of St. Roch in St. Jacob’s Church in a bid to establish ‘religious peace’ and reconcile the Catholics and Philip II with the Protestants.^82^ In this case, the patron saint of the plague sick held a special unifying status in Antwerp.

In the period after 1566, Catholic priests often were ridiculed and also harassed while trying to administer the last sacraments to dying citizens. After the transition to a Calvinist government in 1577, the Calvinist magistrates cracked down on Catholicism, banning priests, confiscating Church goods and removing altars, severely hindering poor Catholics’ access to rituals, sacraments and healing practices.^83^ The confraternity of St. Roch at St. Jacob’s also ceased to function between 1581 and 1585. Jan de Pottre, a merchant and city councillor from Brussels who kept a diary from 1549 onwards, comments that in 1581, Antwerp’s churches were plundered, damaged and closed, masses forbidden and small churches reappointed as grain houses.^84^ Once the city transitioned back to Catholicism, in 1585, the magistrates quickly decreed that the guilds repair the damaged altars. The wardens of St. Jacob’s in 1588 collected donations in the parish to tear down the so-called ‘sea-beggars’, partitions placed in the church by the Protestants in 1580 to shield off the nave from the Catholic altars.^85^

Whereas the shrine of St. Roch had been deployed to bridge religious differences and anxieties, the fear of the spiritual heresy of Lutheranism at times seemed to outweigh any fear of miasma. On occasion, Antwerp’s pre-1577 magistrates described Lutheranism itself in terms of an infection of the populace, when publicly convicting heretics.^86^ Chronicles narrate how, in the years leading up to the 1571 plague outbreak, Catholic priests in the city had ordained that Protestants refusing to take the last sacraments on their deathbed, be condemned to the gallows in the field outside the city. Thus, in 1572, the corpse of a 60-year-old man who refused to confess was allegedly thrown upon a dirt cart and hung on the gallows, ‘as food for the ravens’.^87^ In his chronicle, Godevaert van Haecht, himself a Lutheran, suggests that some Catholics, in a stroke of revenge, met the same fate during the plague outbreak in 1571. They rapidly succumbed to plague and perished without receiving the last sacraments. According to Van Haecht, the priests were now condemned to deny these plague victims burial in consecrated ground as well.^88^ Even Church officials met this fate. On 4 February 1572, according to the *Chronycke van Antwerpen*, a canon of the Cathedral Church, Frans Doncker, perished from plague so quickly that he too could not receive the last sacraments or do confession. ‘Evil heretics’ (i.e. Protestants) immediately jumped at the opportunity and demanded that the priest also be hung on the gallows. This sounds like revenge for the canon’s earlier stance towards Protestants. Indeed, Frans Doncker was said to have exhumed and condemned to the gallows a Protestant woman from the Vlasmarkt, who had died without confessing. Eight days after her burial in the Cathedral cemetery, her body was exhumed and hung on the gallows.^89^

## Mobility and Spatiality: Health Passes and Locked Houses

Not only did Antwerp’s population attempt to protect itself against plague by engaging different spiritual strategies. Its mobility in times of plague, and exposure to it in households, also differed depending on people’s economic and social status that ran along religious fracture lines. In addition to corrupt air and humours, in the sixteenth century, policy makers and medical thinkers viewed the contraction of plague to be related to movement and contact points between people, animals and goods. Plague’s presence thus presented city governments with the dilemma of how to keep up the flow of traffic of goods and people, while reducing the transmission of plague.^90^ This tension between maintaining trade while restricting the movement of people is reflected in two policies established by Antwerp’s government in the years 1570–80: the locking up of the plague sick in their houses, while attempting to facilitate merchants’ and professionals’ mobility by issuing health passes to them—many of whom belonged to the wealthier Protestant class.

Information about the health passes is documented in the Certificatieboek, recording who was able to secure health certificates, their names, professions, contacts and whereabouts.^91^ The registers of the years 1571–2—when Antwerp was still under Catholic rule—contain a batch of 165 requests for passes. From this data, a picture emerges of significant inequalities concerning the mobility of various groups—for the owners of the health passes overwhelmingly belonged to the more affluent segment of Antwerp’s society, dwelling in the old centre.^92^

The earliest known surviving example of a health pass dates to 1484 and was produced in Milan.^93^ Such certificates attested that the place a person had travelled from was free of plague. They were also issued for goods and livestock. Early regional ordinances demanding traders to produce a certificate testifying to the plague-free origins of their goods, survive from nearby Louvain, dating to 1468, Diest in 1469 (thus predating the evidence from Italy) and St. Truiden, in 1515.^94^

The port city of Antwerp itself issued a flurry of certificates related to trade and mobility. No less than 74 registers listing certificates produced in Antwerp have survived for the period 1488–1614.^95^ Most deal with the quality and ownership of goods traded in the region. One of the earliest surviving plague-related health passes for a person from Antwerp dates to 20 September 1509. Symon of Grimbergen of Antwerp and Laureys of Lier, second-hand textile traders (*oudkleerkopers*), obtained a certificate stating that the thirteen sets of beddings they had sold at the Bamis market in Bergen-op-Zoom came from sites free of plague.^96^ In 1514, Antwerp demanded a certificate for all goods coming from infected places.^97^

Cities often coordinated their actions in response to plague—limiting entry into the city by road or boat—with other cities in the region, and in the case of ports, across the sea. Thus, the ordinance to issue health passes in Antwerp, dating to 18 August 1571, came in response to the provision of the mayor and aldermen of the Flemish city of Rijsel (Lille) four days earlier. In reaction, Antwerp ordained that inn-keepers and others were only allowed to offer lodgings to persons and goods with a health certificate in hand.^98^ Earlier, in 1551, the city had forbidden travellers or goods from England to enter Antwerp without due diligence and the presentation of certificates. In the following years, similar provisions were in place for people travelling from Cologne, Frankfurt, Koblenz, ’s Hertogenbosch, Delft and Zierikzee (1553), Paris and other cities in France (1562), England and France (1563), Hamburg and Arnhem (1565), Turnhout (1571) and London (1593).^99^ Between 1567 and 1576, moreover, the city asked for certificates to check a person’s military allegiance.^100^ In 1596, Antwerp introduced an inspection at the gates, demanding that soldiers carry passports, while expelling all beggars—an indication of how public health crises can strengthen the maintenance of boundaries.^101^ However, there is no indication that documentation was used to check the movement of Calvinists in and out of the city.

To whom did the city extend health passes between 1571 and 1572? In total, the urban authorities issued 165 certificates between August and November 1571 to 189 (co-)applicants, five of which were applied for via an intermediary. In 108 cases, the passes identified the person with whom the applicant declared to have been in contact. Most of the certificates were produced in August 1571, at the height of the plague outbreak. Almost half of the applicants came from Ghent. Finally, whereas the health passes examined by Alexandra Bamji predominantly were presented to men (barely 2 per cent were issued to women), a slightly higher number of certificates (85 per cent for men, 10 per cent for women and 4 per cent for both husband and wife) were issued to women in Antwerp.^102^

Not only did many applicants come from other cities, over half had professions involving the mobility of goods and persons: 35 were merchants, 13 worked in the textile industry, 6 in the food trade, 5 as porters and another 5 were seamen (64 in total). City officials, including the clerk of the secretary, and health professionals, such as the plague doctor, barber and apothecary, also applied for passes. In sum, the applicants living in Antwerp had a specific socio-professional profile, worked in trade or public health, and belonged to the wealthier social and most likely predominantly Protestant echelons.^103^ The health certificates issued to Calvinists and Lutherans in the early 1570s attest their economic and political influence in a Catholic city and their ability to access administrative resources—and thus also perhaps suggest a level of accommodation on the part of the Catholic magistrates, eager to keep their trade.

**Fig. 5.**
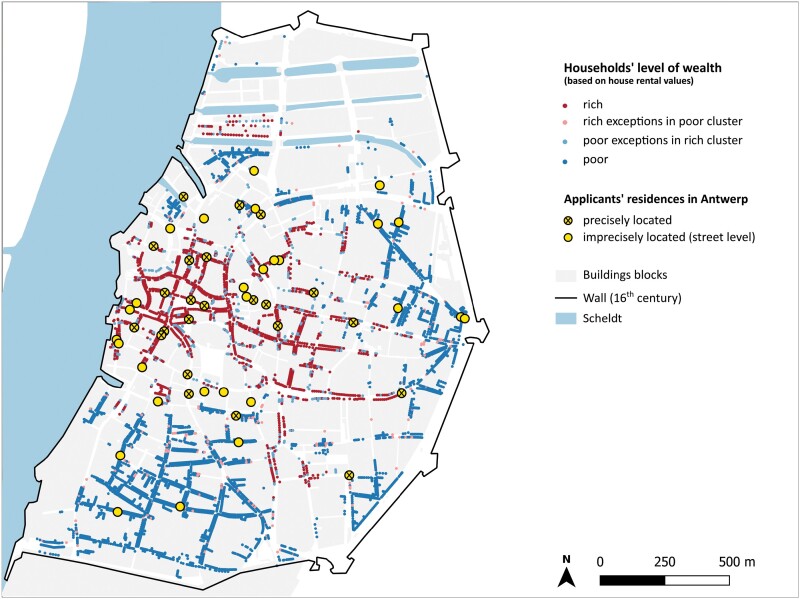
Residences of the health pass applicants living in Antwerp in 1571 (51 residences) and information about the households’ social stratification (based on clustering of house rental values in 1580)

The socio-religious profile of recipients of Antwerp’s health passes is strengthened from a spatial perspective, if we look at their places of residence (Figure 5).^104^ Significantly, most of the applicants lived or were connected to households in the most affluent, Protestant neighbourhoods of the city, such as around the Cathedral, near to the city hall, and on the main market square (Grote Markt). Among the applicants who lived in Antwerp, a large majority of them dwelled within the twelfth-century fortifications, the oldest part of the city, while only a few lived in the poorer western or southern neighbourhoods.

Besides health passes enabling the movement of merchants and officials, another, more restrictive standardised procedure took shape in the sixteenth century in Antwerp: locking infected persons in their homes as a form of quarantine. As we can see in Figure 8, whereas the affluent inhabitants of Antwerp’s city centre managed to apply for health passes, the households that were locked lay predominantly in the city’s poorer Catholic districts. This raises the question why so. As discussed below, two factors seem relevant: firstly, the inequality in financial status and the spatial dimensions of the properties in the various districts, and, secondly, social status and class bias.

Before looking in more detail at the religious, spatial and socio-economic profiles of the sealed households, it is important to trace the steps taken in the process of locking houses, to properly understand the data on the closed houses recorded in the locksmith’s register between 1579 and 1580—the period under Calvinist rule. From the late fifteenth century, quarantine at the household level was a common practice both in this city and elsewhere in the Low Countries. When a member of a household became infected with plague, their housemates immediately had to attach a bundle of straw (*stoewisch*) above the door or high up on the windowsill, for a period of 6 weeks.^105^ This custom may stem from the practice of laying the dead on straw, to avoid further contamination of bed sheets.

In Antwerp, however, the regulations concerning quarantine become more complex in the 1550s. Its extensive 1557-plague ordinance is a perfect example of the quarantine measures taken in the city—the same rules are continuously reaffirmed over the next five decades.^106^ In 1557, Antwerp’s authorities specified the options people had when plague was detected—although it remains unclear how and by whom the diagnosis was made. So long as no one had died or was unable to walk, people had the option of temporarily abandoning their homes and moving into hospitals, or leaving the city altogether. Upon leaving, they had to lock *and* seal off their houses with a chain. They then had to report their departure to the *keurmeesters,* in person or through friends or neighbours, and declare on which day the disease had entered their homes, which was registered on that same day.^107^ Moreover, ‘to ensure that the house will not be opened earlier than ordained’, the city’s locksmith had to attach an additional lock to the door or gate, of which the *keurmeesters* held the keys.^108^ If a person died, or wanted to stay and take care of patients, or simply preferred to rest inside, they in all events had to remain locked in, and to ‘refrain from being among people’.^109^ Locking infected houses was practised in cities across the Alps as well, such as in Florence and in Verona, where the households of San Zeno were sealed off preventively, as they were in Milan during the 1575–8 outbreak and in Geneva.^110^

In 1571, the city’s policy became stricter, however. People moving out of the infected houses were not allowed to stay at other residents’ private homes. Instead, they were obligated to go ‘to the hospitals and other specially appointed places, or leave the city’.^111^ The city magistrates lamented that people were not obeying their orders, however, and continued to mingle with other people or move through the city, ‘which is a major cause of the rapid spread of the disease’.^112^ The magistrates decided that from then on, ‘if people wish to stay in their homes, these homes shall be locked from the outside so that no one can move in or out, other than the confessor *(biechtvadere*), plague master or surgeon, *cellebroeder* or *Swarte Suster*’.^113^ In that same year, the chronicles observed citizens throwing stones at the *cellebroeders* in the streets and at the gates of the monastery, suggesting animosity because of the *cellebroeders’* complicity.^114^ To address the issue of how to get food and drink to the people locked in their houses, the authorities decreed that ‘people assigned by the city’ would deliver these necessities to the locked houses, keeping account of the expenses made—these are the *toesienders* mentioned above.^115^ People locked in were visited twice a day and given food by lowering a basket from the first floor. They were only allowed to open their windows to air their homes between midnight and four in the morning.^116^

The resident poor, predominantly belonging to the Catholic segment of the population, were also the target of more specific measures. The city’s poorest inhabitants (*aerme schamel lieden*), exposed to substandard living conditions, were not allowed to stay in their homes at all. The *aelmoezeniers* and *keurmeesters* deemed it better, so the ordinance declared, ‘to prevent further danger, to oblige them to leave’.^117^ The evicted sick had to apply through the aforementioned officials to be allowed to stay at a hospital. Some of the hospitals, which usually hosted itinerant poor and travellers for a few days, were converted to house Antwerp’s resident poor plague patients and members of their households for several weeks, as was common in cities elsewhere too, especially in Italy.^118^ The fact that the Calvinist government did not abolish these rules, first issued in 1571 under Catholic rule, suggests it continued to run a plague hospital in order to be able to enforce measures such as eviction of the poor from their homes. The efforts undertaken in 1580 and 1581, discussed above to improve the capacity of plague facilities, albeit not fully realised, echo this interest.

**Fig. 6.**
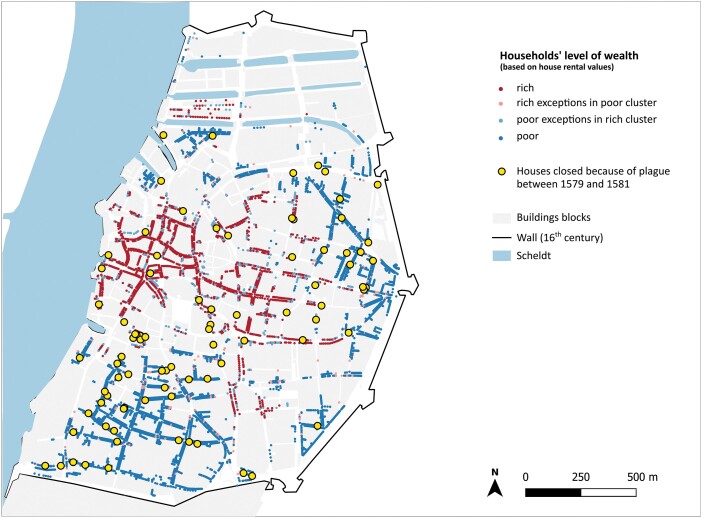
Overlay of the spatial distribution of houses closed because of plague between 1579 and 1580 and information about the households’ social stratification (based on clustering of house rental values)

Turning now to the locksmith’s register of 1579–80, this document contains a total of 116 entries, with specific data on streets and sometimes even houses, and dates of closure and reopening, allowing us to map this information (Figure 6).^119^ There is a striking variability in dates of closure and reopening. On average, the houses were closed for 6 weeks, which was fully in line with the policies prescribed in the plague ordinances. However, the rules were often broken. In fact, 25 per cent of the houses were closed for less than 5 weeks. Another 25 per cent were closed for more than six and a half weeks. One house was closed just for four days, while another remained locked for no less than 119 days. The reason is not stated.

The records also give us insight into the potential spread of the disease across the city. John Henderson suggested that the plague epidemic in Florence in 1630–3 arrived from the north along the route to Bologna, entering the gate and then spreading inwards.^120^ A spatial reconstruction of the months in which houses were locked in Antwerp, nonetheless, does not show a clear pattern of consecutive hotbeds of infection in certain areas (a video of the closing and opening of houses is available here). Most of the earlier cases are more towards the city’s periphery, but this pattern is relatively weak.

There is, however, a clearer correlation between levels of wealth and poverty expressed in real estate values and the occurrence of house closures. If we compare the locked houses with data on house rental values, it becomes evident that relatively fewer houses were locked in the areas with the highest real estate prices (Figure 6). A horizontal axis of high-value homes running through the centre of the city, where many prominent economic activities took place, is strikingly void of locked homes. Significantly, the district with few locked houses was an overwhelmingly Protestant-leaning neighbourhood. This discrepancy can be explained in various ways. A likely explanation is that fewer wealthier households became infected because of larger household sizes, and because the inhabitants had more options to leave the city during outbreaks. Another suggestion is class bias in policing practices: under Calvinist rule, the well-to-do and Protestants may have been able to prevent detection of plague to avoid closure. Nonetheless, some houses belonging to wealthy property owners in the less affluent, more densely built-up neighbourhood to the south of the old centre, in the parish of St. Andries, were locked as well.

**Fig. 7.**
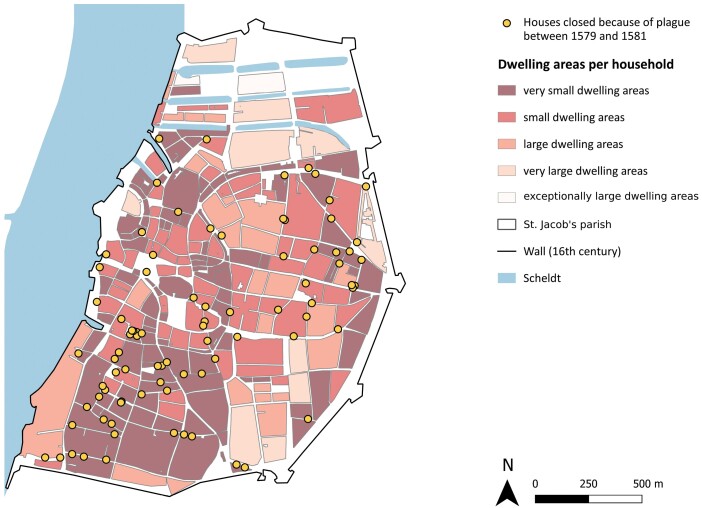
Spatial distribution of closed houses and approximation of the size of dwelling areas per household

The number of locked houses is, on aggregate, higher in areas with smaller dwelling spaces.^121^ Beyond a possible class bias in policing, this, unsurprisingly, suggests that living in more cramped spaces, with a higher population density, increased the risk of being exposed to plague. The levels visualised in various intensities of red in Figure 7 show the density of households per islets, not per building. Certainly, the old centre or St. Andries parish was densely populated and built up as well, but the more well-to-do people residing there could afford less cramped inside living spaces. Nonetheless, as Figure 7 shows, exceptionally large dwelling areas were not completely exempt from sealing off either.

**Fig. 8.**
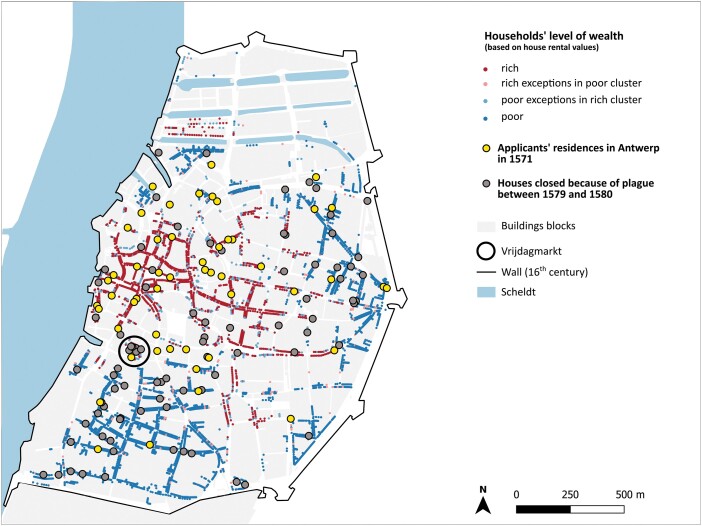
Spatial distribution of houses closed because of plague and residences of the health pass applicants, overlayed with information about the households’ social stratification (based on clustering of house rental values in 1580)

Finally, including both data sets in one map (Figure 8) confirms the discrepancy between the social groups targeted by the closure of houses and the recipients of the health passes. The locksmith’s registers indicate that a relatively high number of households were locked around the flea market called the Vrijdagmarkt (Figure 8) in 1579. Nowhere else in the city was there such a high cluster of closures due to plague. This market emerged in the 1550s as a hub where second-hand textiles and goods exchanged hands. At least three of the sealed houses on the Vrijdagmarkt are known to have belonged to second-hand traders (*oudkleerkopers*). The market space was, accordingly, a site of specific concern in the city authorities’ eyes, for it attracted a lot of traffic and, importantly, potentially infected wares. Indeed, physicians considered textiles from infected households to release poisonous vapours spreading disease.^122^ Catholics and Protestants were almost equally represented among this group of traders.^123^

More generally, a statistical exploration (multiple correspondence analysis) conducted on the variables (all per ward or quarter—*kwartier*) described above, namely number of locked houses; number of health passes per applicant’s residences; percentage of Catholics; number of wealthy and poor households; approximate size of dwelling areas; reveals no clear correlations between them.^124^ However, some recurring similarities in the statistics are evident: *kwartiers* with a high number of poor households, small dwelling areas and high percentages of Catholics tend to have more locked houses, but there are exceptions.

**Fig. 9:**
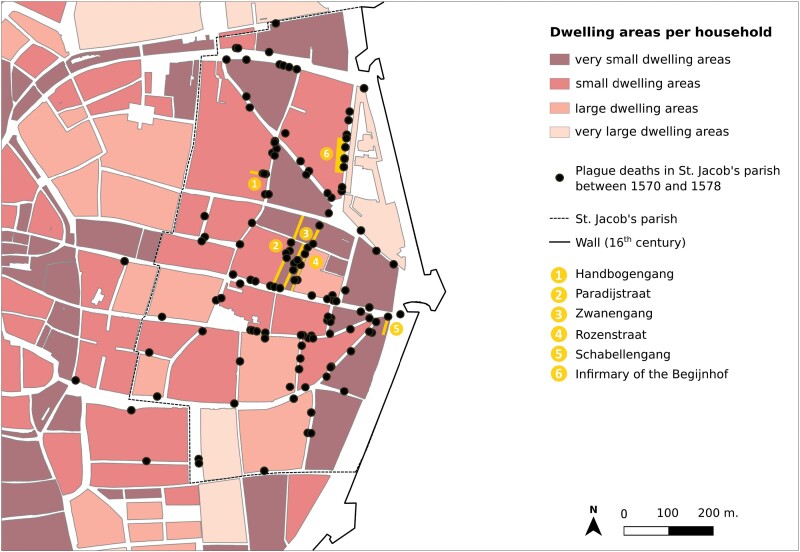
Overlay of plague mortality and approximation of the size of dwelling areas per household

Ann Carmichael and Samuel K. Cohn Jr. both have argued that the poor in Italy contended with a higher vulnerability to plague, although this was not necessarily the case during the first outbreaks of plague in the fourteenth century.^125^ In Antwerp’s St. Jacob’s parish records, the concentration of deaths in the more narrow streets and alleys called Handbogengang, Paradijstraat, Zwanengang, Rozenstraat, and Schabellengang (Figure 9),^126^ similarly suggest a connection between plague, socio-economic status and housing conditions.^127^ St. Jacob’s parish was located in the north-east of Antwerp. It numbered about 2,500 households, home to approximately 12,500 people.^128^ This is an area dominated by Catholic dwellings. The circulation of the disease shows up, for example, in a cluster of deaths around the corner of the Drie-kandelarengang and the St. Jacobsmarkt recorded in the parish death register. It is conceivable that the living and physical conditions of the poorest residents, dwelling mainly in the small alleys, rendered them particularly vulnerable to plague. Yet another risk factor may be the traffic and contacts with other people outside their houses in the narrow streets. Indeed, the ‘centre’ or middle of the parish, with comparatively smaller dwelling areas, seems to have been strongly hit by plague deaths.

A large portion of the St. Jacob’s parish was poor and dominated by Catholics, thus confirming the convergence of socio-economic stratification and religious denomination. In sum, in these poorer, Catholic areas, population density per household was higher—another factor increasing the chance of infection with plague.

In addition to housing conditions—size, composition of members—profession and tasks assigned also increased a person’s susceptibility to contract the disease. Plague, like dysentery and typhus, spread in households.^129^ Cohn has argued that plague victims in the same household tended to die within a remarkably short time span.^130^ Daniel Curtis, and earlier Carole Rawcliffe, raised the question whether higher female mortality levels might be related to expectations of care inside the household and in public health institutions, in addition to access to resources and protection.^131^ There are instances known of women taking in young, sick girls to care for them.^132^ However, no clear relation exists between space, household, and gender in the St. Jacob’s data: there is a higher fatality rate among women and multiple fatalities in households, but these are not necessarily related to space. Women also worked as cleaners (*schrobsters*) in larger households.^133^ In Antwerp, however, men also clearly carried out various health tasks involving physical and spiritual care, acting as *pestmeesters*, *cellebroeders* and cleaners. Some of these male health workers also died from plague. The parish priest and the sexton of Antwerp’s Cathedral were among the victims who perished in 1571, possibly as a result of heightened exposure as spiritual carers.^134^

Finally, in several instances in St. Jacob’s parish, entries show that people perished from plague in the streets. This is visualised in the retable in St. Jacob’s parish church (Figure 4), which depicts a man succumbing to the disease outside. Does this suggest the disease was so deadly that people were too weak to return to their homes? Or was this a deliberate strategy, to avoid infecting other household members or forcing them into quarantine? Some of the victims died in houses dedicated to caring for the sick. The cobbler Jan van de Hoek, for instance, died in the house of the *cellebroeders* on 22 September 1575. Yet only two people, a man and a woman from Nieuwstad, are registered as having died at the plague hospital, a year earlier in September 1574. Moreover, at least five people were recorded to have died in the Rodestraat, where the Beguines’ *infirmerie* was housed—yet the records do not specify whether they died in that specific institution.

## Conclusion

Amidst the challenges of religious struggle and conflict in sixteenth-century Antwerp, plague was an extra, destabilising factor. While mortality rates did not cause a collapse in urban population numbers—these were at least in the first half of the sixteenth century more than compensated for by mass migration into the city—a closer look at the range of available sources reveals ongoing dealings with plague. The sections above have sought to trace Antwerp’s wide range of interactions. The city’s magistrates were highly aware of plague threats and reactive to what was going on in the city, and to the complaints and concerns of inhabitants—at least those with social and political influence.

The case of Antwerp highlights how Catholics and Protestants’ susceptibility to and experience of plague were different. They had access to or preferred different forms of spiritual care. After the Calvinist take-over of government in 1577, Catholics were deprived of the prophylactic tool of processions. Calling upon the intercession of the saints, or organising special processions, was halted temporarily, which presumably caused anxiety among the Catholic population. Such restrictions might have had a spiralling effect, given that anxiety itself was considered, in medical terms, an important factor in weakening a person’s physical stamina.

The variance in the effects of plague was, we suggest, shaped by a combination of preventive (politically and economically motivated) measures on the one hand, and the spatial and socio-economic composition of infected households on the other, which were clustered along religious lines. Spatiality, mobility, class and religion are thus key interlinking factors determining the differentiated, unequal impact of plague in Antwerp. This article thus underscores the relevance of spatial-environmental conditions in public health. Most notably, we found that the size of the dwelling area in combination with the number of persons in a household influenced the occurrence of plague, as suggested by the data on the locked houses and of St. Jacob’s parish. Cramped living spaces, which were more common among the poorer social echelons (and perhaps also migrant populations), meant higher recorded infection rates. However, these rates were perhaps skewed by the biased gaze and policing by the authorities.

Such were the complexities of dealing with plague, that touched the lives and homes of so many people in Antwerp. Protestants, residing in the wealthier district, in less cramped houses, had more options to avoid the spread of plague and continue to go about their daily business, aided by health passes. The velocity of plague disrupted the burial practices that were already contested in political-religious struggles. Plague thus cut through many different spheres: social, economic, religious, through space and via movement, with different outcomes for a diverse population.

## Footnotes

1 R. Fagel, ‘The Origins of the Spanish Fury at Antwerp (1576): A Battle Within City Walls’, *Early Modern Low Countries*, 2020, 4, 102–123; G. Parker, *The Dutch Revolt* (London: Allen Lane, 1985), 178, 190.

2 G. Marnef, *Antwerpen in de tijd van de Reformatie: Ondergronds protestantisme in een handelsmetropool 1550–1577* (Amsterdam/Antwerp: Meulenhoff, 1996), 268–273.

3 Recently mentioned in M. Pye, *Antwerp: The Glory Years* (London: Penguin Books, 2021).

4 This article concentrates on different Christian groups and does not focus on comparisons with the Jewish population.

5 The bacterium *Yersinia pestis* spread in micro-environments and was hosted by a range of animals. Monica Green argues that four branches of it emerged in the later Middle Ages (the ‘Big Bang’). Animals such as marmots and human actors facilitated its spread, in the Second Plague Pandemic possibly through grain shipments by the Mongols in the thirteenth century. See M.H. Green, ‘The Four Black Deaths’, *American Historical Review*, 2020, 125/5, 1601–1631; M.H. Green, ‘Editor’s Introduction to Pandemic Disease in the Medieval World: Rethinking the Black Death’, *The Medieval Globe*, 2015, 1, 9–26.

6 Stadsarchief Antwerpen, 108: *Kronijk van Jan van Wesenbeke*, fol. 113.

7 Godevaert van Haecht, *Kroniek over de troebelen van 1565 tot 1574 te Antwerpen en elders*, ed. R. van Roosbroeck, 2 vols (Antwerpen: De Sikkel, 1929–1930), II, 152. Godevaert was not residing in Antwerp at the time, however.

8 The variance depends, among other things, on the inclusion of foreign merchants in the population numbers. Stadsarchief Antwerpen, Insolvente Boedelkamer, IB3017: Insolvente Boedel Gilbert van Schoonbeke—‘sommieren overslach’.

9 A.F.C. van Schevensteen, ‘Over pestepidemieën te Antwerpen in vroeger tijden’, *Verslagen en mededelingen van de Koninklijke Vlaamse Academie voor Taal- en Letterkunde 1932* (Ghent, 1932), 1055–1092, 1065–1067.

10 Some cities experienced high mortality rates in later centuries as well, such as Florence and London. See J. Henderson, *Florence Under Siege: Surviving Plague in an Early Modern City* (New Haven: Yale University Press, 2019); G. Alfani, ‘Plague in seventeenth-century Europe and the decline of Italy: An epidemiological hypothesis’, *European Review of Economic History*, 2013, 17/4, 408–430, for a higher impact in Italy in the seventeenth century. See R. Ladan, *Gezondheidszorg in Leiden in de late middeleeuwen* (Hilversum: Uitgeverij Verloren, 2012); L. Noordegraaf and G. Valk, *De gave Gods: De pest in Holland vanaf de late middeleeuwen* (Bergen, NH: Octavo, 1988) for outbreaks of plague in the Low Countries. An important revision of early mortality rates in the Low Countries in D.R. Curtis and J. Roosen, ‘The ‘Light Touch’ of the Black Death in the Southern Netherlands: An Urban Trick?’, *The Economic History Review*, 2019, 72/1, 32–56, arguing that the plague struck the Low Countries more severely than previously thought.

11 A.F.C. van Schevensteen, *Documents pour servir à l’étude des maladies pestilentielles dans le marquisat d’Anvers jusqu’à la chute de l’Ancien Régime*, 2 vols (Brussels: Lamertin, 1931–1932), i, 192.

12
*Ibid*., 198.

13 A.K.L. Thijs, *Van Geuzenstad tot katholiek bolwerk: Maatschappelijke betekenis van de Kerk in contrareformatorisch Antwerpen* (Turnhout: Brepols, 1990), 10.

14 Between September and November 1585, the new magistrate conducted an inquiry among the civil guard and members of the armed guilds to detect and expel unreliable (read: especially Calvinist) members. The representativeness of this census for the Antwerp population is explained in R. Boumans, ‘De getalsterkte van Katholieken en Protestanten te Antwerpen in 1585’, *Revue belge de philologie et d’histoire*, 1952, 30/3–4, 741–798. J. Van Roey, ‘De sociale structuur en de godsdienstige gezindheid van de Antwerpse bevolking op de vooravond van de reconciliatie met Farnèse (17 augustus 1585)’ (unpublished dissertation, University Ghent, 1963), 191. R. van Kooten, ‘Antwerpen 1584. Oorlog en ongelijkheid in de stedelijke ruimte’ (unpublished dissertation, Universiteit Antwerpen, 2021), 278-81. The last study presents a detailed geographical analysis of religious and social segregation.

15 Thijs, *Van Geuzenstad*, 14.

16 R.K. Rittgers, ‘Protestants and Plague: The Case of the 1562/63 *Pest* in Nürnberg’, in Franco Mormando and Thomas Worcester (eds), *Piety and Plague: From Byzantium to the Baroque* (Kirksville: Pennsylvania State University Press, 2008), 132–155.

17 L.J. Rather, ‘The “Six Things Non-Natural”: A Note on the Origins and Fate of a Doctrine and a Phrase’, *Clio Medica*, 1968, 3/4, 337–347; L. García-Ballester, ‘On the Origins of the Six Non-Natural Things in Galen’, in *Galen und das hellenistische Erbe: Verhandlungen des IV. Internationalen Galen-Symposiums veranstaltet vom Institut für Geschichte der Medizin am Bereich Medizin (Charité) der Humboldt-Universität zu Berlin 18–20. September 1989*, ed. J. Kollesch and D. Nickel (Stuttgart: Franz Steiner Verlag, 1993), 105–115.

18 For the significance of music, singing and dance to protect against the plague, informed by the Platonic concept of sympathetic resonance with a cosmic world soul bringing order and life to the material world, see R. Chiu, *Plague and Music in the Renaissance* (Cambridge: Cambridge University Press, 2017); R. Chiu, ‘Singing on the Street and in the Home in Times of Pestilence: Lessons from the 1576–78 Plague of Milan’, in *Domestic Devotions in Early Modern Italy*, ed. M. Corry, M. Faini, A. Meneghin (Leiden: Brill, 2018), 27–42; G. Rohmann, ‘Tanz als Krankheit, Tanz als Therapie. Die Formierung eines religiös-medizinischen Konzepts (15. und 16. Jahrhundert)’, *Das Mittelalter*, 2018, 23/2, 281–307.

19 Rittgers, ‘Protestants and Plague’. See also for the continuance of the reliance on natural healing: A. Walsham, *The Reformation of the Landscape: Religion, Identity, and Memory in Early Modern Britain and Ireland* (Oxford: Oxford University Press, 2011). For responses in the Islamic world: N. Varlik (ed.), *Plague and Contagion in the Islamic Mediterranean: New Histories of Disease in Ottoman Society* (Kalamazoo: Arc Humanities Press, 2017).

20 P. Rieder, ‘Miracles and Heretics: Protestants and Catholic Healing Practices in and around Geneva 1530–1750’, *Social History of Medicine*, 2010, 23/2, 227–243.

21 C. Rawcliffe, *Urban Bodies: Communal Health in Late Medieval English Towns and Cities* (Woodbridge: Boydell, 2013); A. Agresta, ‘From Purification to Protection: Plague Response in Late Medieval Valencia’, *Speculum*, 2020, 95, 371–395; K. Wilson Bowers, *Plague and Public Health in Early Modern Seville* (Rochester, NY: University of Rochester Press, 2013).

22 A.G. Carmichael, *Plague and the Poor in Renaissance Florence* (Cambridge: Cambridge University Press, 1986); Henderson, *Florence Under Siege*, 3–4.

23 S.K. Cohn, *Cultures of Plague: Medical Thinking at the End of the Renaissance* (Oxford: Oxford University Press, 2010), 292–293. B. Pullan, ‘Plague and Perceptions of the Poor in Early Modern Italy’, in T. Ranger and P. Slack (eds), *Epidemics and Ideas: Essays on the Historical Perception of Pestilence* (Cambridge, 1992), 101–123, who distinguishes between the poor as beneficiaries, victims and bearers of plague. See also G. Calvi, *Histories of a Plague Year: The Social and the Imaginary in Baroque Florence* (Berkeley: University of California Press, 1989); C.M. Cipolla, *Fighting the Plague in Seventeenth-Century Italy* (Madison: University of Wisconsin Press, 1981); Z. Blažina-Tomić and V. Blažina, *Expelling the Plague: The Health Office and the Implementation of Quarantine in Dubrovnik, 1377–1533* (Montréal, CA: McGill-Queen’s University Press, 2015).

24 J.L. Stevens Crawshaw, *Plague Hospitals: Public Health for the City in Early Modern Venice* (Farnham: Ashgate, 2012). A good overview of studies of mortality rates in D.R. Curtis and J. Roosen, ‘The Sex-Selective Impact of the Black Death and Recurring Plagues in the Southern Netherlands, 1349–1450’, *American Journal of Physical Anthropology*, 2017, 164, 246–259. For recent revisions in pathology see M.H. Green, ‘The Four Black Deaths’, *The American Historical Review* 2020, 125, 1601–31.

25 On plague in the Dutch Republic see A.H.M. Kerkhoff, *Per imperatief plakkaat: Overheid en pestbestrijding in de Republiek der Verenigde Nederlanden* (Hilversum: Verloren, 2020), who however argues that few regulations were issued before the seventeenth century.

26 W. Dupont, ‘“Sie eens ’t verzwakte volk”: De bestrijding van pest in Antwerpen tijdens de vijftiende en zestiende eeuw’, *Stadsgeschiedenis*, 2007, 2/2, 85–104, at p. 89 records 62 outbreaks of plague between the years 1439 and 1599, of which the years 1571–1572, 1575–1586, 1588–1597 and 1599 are especially relevant here. The plague in Antwerp is mentioned briefly in J. Hendricx, ‘Het dode gevaar? Begraven en Hygiëne te Antwerpen van de 16e tot de 18e eeuw’, in W. Pottier and P. Maclot, eds., *’n Propere tijd!?: (On)leefbaar Antwerpen thuis en op straat (1500–1800)* (Antwerp: Antwerpse Vereniging voor Bodem- en Grotonderzoek, 1988), 26–37; P. Poulussen, *Van burenlast tot milieuhinder: Het stedelijk leefmilieu, 1500–1800* (Kapellen: DNB/Pelckmans, 1987).

27 Van Schevensteen, *Documents*. Van Schevensteen did not publish the parochial register of deaths starting in 1568, however.

28 Stadsarchief Antwerpen, Register van zieke en begraven parochianen.

29 Stadsarchief Antwerpen, Certificatieboek 1571–1572. Printed in Van Schevensteen, *Documents*, I, 101–123.

30 Stadsarchief Antwerpen, Pesthuizen en pestzieken. Printed in Van Schevensteen, *Documents*, I, 176–189.

31 The data on plague can be compared with the information from the Gilbert Degueldre cadastre of 1584, which gives valuable insight into the socio-economic position of Antwerp’s households G. Degueldre, *Kadastrale ligger van Antwerpen (1584–1585): Proeve van reconstructie op de vooravond van de scheiding der Nederland*en, 14 vols (Antwerp: Felix Archief, 2011). See also Van Kooten, ‘Antwerpen 1584’. Household poverty and wealth are related to the median classes of the rental values of the houses in which they lived: <½ median = very poor; ½ median–median = poor; median–2x median = middle class; 2x–4x median = rich; >4x median = very rich. This is based on comparable research by Johan Dambruyne on Ghent in the same period; see J. Dambruyne, *Corporatieve middengroepen: Aspiraties, relaties en transformaties in de 16de-eeuwse Gentse ambachtswereld* (Gent: Academia Press, 2002), XXVIII, 366–367. The outcome of a Cluster and Outlier analysis shows the spatial clustering of rich (red) and poor (blue) households perfectly in line with these median classes (see Figures 2, 5, 6 and 8).

32 Van Kooten, ‘Antwerpen 1584’, 158. A similar study mapping plague in Dijon is P. Galanaud, A. Galanaud, and P. Giraudoux, ‘Historical Epidemics Cartography Generated by Spatial Analysis: Mapping the Heterogeneity of Three Medieval “Plagues” in Dijon’, *PLoS One* 2015, 10/12: e0143866, https://doi.org/10.1371/journal.pone.0143866.

33 In the course of the fifteenth century, the old *Bourse* no longer adequately served its purpose, and in 1531 the authorities ordered the construction of a new building just outside the old centre, beyond the original town defence structures.

34 A calculation of the correlation between average house rental value per neighbourhood and the share of Catholic households leads to a significant negative relationship of −0.403 (p<0.01). Van Kooten, ‘Antwerpen 1584’, 281 shows support for the so-called ‘Law of Van Roey’ that in general holds that the share of Catholics decreased as welfare rose.

35 Van Kooten, ‘Antwerpen 1584’, 89-91.

36 FAA: GA 4830 en 4617/ Databank G. Marnef.

37 Stadsarchief Antwerpen (Felix Archief), Gebodboeken, BE SA 174775, A-E (1489–1612). These manuscripts are published in calendar form in ‘Index der Gebodboeken 1489–1620, ed. P. Genard, *Antwerpsch Archievenblad*, 1864, 1, 120–464.

38 A.F.C. van Schevensteen, ‘De hygiënische maatregelen van het magistraat van Antwerpen in de vijftiende eeuw’, *Nederlands Tijdschrift voor Geneeskunde*, 1927, 71, 2479–2492.

39 The chronicles about Antwerp dealing with the period after 1585 do not comment on the outbreaks of plague in 1597 and 1601.

40 B. Blondé and J. Puttevils (eds.), *Antwerp in the Renaissance* (Brepols: Turnhout, 2020).

41 M. Dijkdrent, ‘Healing Through Space: An Essay on the Meanings of Space in the St. Caeciliagasthuis in Leiden’ (unpublished MA thesis, Leiden University, 2020).

42 R. MacKay, *Life in a Time of Pestilence: The Great Castilian Plague of 1596–1601* (Cambridge: Cambridge University Press, 2019).

43 Van Schevensteen, *Documents*, I, 156.

44
*Ibid.*, 93, 161.

45
*Ibid.*, 81–98, 151–164.

46 A considerable number of the ordinances were regular calls to inhabitants to clean certain spaces, such as streets and waterways.

47 Tabulating the ordinances concerning public hygiene and comparing them with plague years in Antwerp, it is evident that the organisation of urban sanitation was not so much a reaction to outbreaks of plague, but rather a regular and integrated component of urban governance. G. Geltner, ‘The Path to Pistoia: Urban Hygiene Before the Black Death’, *Past & Present*, 2020, 246, 3–33. For urban sanitation beyond outbreaks of plague: J. Coomans, *Community, Urban Health and Environment in the Late Medieval Low Countries* (Cambridge: Cambridge University Press, 2021).

48 Van Schevensteen, *Documents*, I, 153.

49
*Ibid*., 79–80. *Werken van barmhartigheid: 650 jaar Alexianen in de Zuidelijke Nederlanden* (Louvain: Stedelijk Museum Vander Kelen-Mertens, 1985); S.M.C. Leupen, ‘De kloosters van de cellebroeders en -zusters in het graafschap Holland en Zeeland tot aan de Reformatie’, *Historisch Tijdschrift Holland*, 1998, 30/2 63–92; C.J. Kauffman, *Tamers of Death: The History of the Alexian Brothers* (New York: The Seabury Press, 1976).

50
*Ibid.*, 6: ‘dat men van nu voordaene, gheene siecke van der pestilencien meer leggen en sal op de vesten der stadt, ende dat de ghene, dier leggen men van daer sal verlogeren’.

51 Van Schevensteen, ‘Over pestepidemiën te Antwerpen’, 1059–1060.

52 Van Schevensteen, *Documents*, I, 58–59; P. De Commer, ‘Hygiëne en gezondheidszorg in Antwerpse gezondheidsinstellingen tijdens het Ancien Régime’, in P. Maclot and W. Pottier (eds.), *‘N Propere tijd?! (On)leefbaar Antwerpen thuis en op straat* (Antwerp: Antwerpse Vereniging voor Bodem- en Grotonderzoek, 1988), 37–44, at 40.

53 Van Schevensteen, ‘Over pestepidemiën te Antwerpen’, 1067.

54 De Commer, ‘Hygiëne en gezondheidszorg’, 40–42.

55 Van Schevensteen, ‘Over pestepidemiën te Antwerpen’, 1067.

56 Van Schevensteen, *Documents*, I, 203–205.

57 Crawshaw, *Plague Hospitals,* 22–25; A.G. Carmichael ‘Plague Legislation in the Italian Renaissance’, *Bulletin of the History of Medicine*, 1983, 57/4, 508–525 at 513–521.

58 A.G. Carmichael, ‘Contagion Theory and Contagion Practice in Fifteenth-Century Milan’, *Renaissance Quarterly*, 1991, 44/2, 213–256.

59 When health boards did emerge, moreover, it focused more on policing the health professionals rather than advising policy makers. Coomans, *Community, Urban Health and Environment*, 245-46.

60 Van Schevensteen, *Documents*, I, 58–59 is one of the earlier very elaborate versions of the regulations. See also 84–85.

61 Around 1600, the term *keurmeesters* was gradually replaced by the term *policycamer*, where the *opperkeurmeesters* were in charge.

62 In the city accounts (*stadsrekeningen*), we find records of the reimbursement of medical personnel specialised in plague. See for instance Van Schevensteen, *Documents*, I, 26, 34, 38, 42, 166, 195.

63
*Ibid*., 92.

64
*Ibid*., 153–159.

65
*Ibid.*, 196.

66 Rekening fol. 157v.; Van Schevensteen, *Documents*, I, 145. Cf. Cohn, *Cultures of Plague*, 104–105 for the locking up of people in their houses during Milan’s outbreak of 1575–1578.

67 Ordinance of 27 June 1555 (Van Schevensteen, *Documents*, I, 55, 60), repeated on 3 August 1571 (Van Schevensteen, *Documents*, I, 87), decreeing that infected houses should be locked by the inhabitants when they left, and the neighbours and *keurmeesters* notified.

68 Van Schevensteen, *Documents,* I, 206.

69 See for instance *Ibid*., 314 (1611).

70
*Ibid*., 281. In 1571, the Antwerp magistracy had implicated these cleaners in a conspiracy theory that was already circulating in the 1460s and 1520s in Brabant, in 1530 in Geneva and in France (by *engresseurs*) between the 1530s and 1580s in the context of tensions between Protestants and Catholics. Male and female *reeuwers* and *schrobbers* in Brabant were accused of smearing grease from plague houses on the doorknobs of the houses of the wealthy. Accusations of greasing were sometimes combined with complaints of heresy, witchcraft and satanic pacts, which in the early seventeenth-century also surfaced in Milan. See W.G. Naphy, *Plagues, Poisons and Potions: Plague-spreading Conspiracies in the Western Alps c. 1530–1640* (Manchester: Manchester University Press, 2002). S.K. Cohn Jr., *Epidemics: Hate and Compassion from the Plague of Athens to Aids* (Oxford: Oxford University Press, 2018), 131–160.

71 Van Schevensteen, *Documents*, I, 265.

72 Cohn, *Cultures of Plague*; Henderson, *Florence Under Siege*, 13.

73 Agresta, ‘From Purification to Protection’; I. Fenlon, *The Ceremonial City: History, Memory and Myth in Renaissance Venice* (London: Yale University Press, 2007), 224; N. Archambeau, *Souls Under Siege: Stories of War, Plague and Confession in Fourteenth-Century Provence* (Ithaca: Cornell University Press, 2021).

74 L. Marshall, ‘Confraternity and Community: Mobilizing the Sacred in Times of Plague’, in B. Wisch and A. Cole Ahl, eds, *Confraternities and the Visual Arts in Renaissance Italy: Ritual, Spectacle, Image* (Cambridge: Cambridge University Press, 2000), 20–45.

75 M.-L. Leonard, ‘Wounded: Healing Communal Wounds: Processions and Plague in Sixteenth-Century Mantua’, *Science Museum Group Journal*, 2019, 11. https://dx.doi.org/10.15180/191106/001.

76 Van Schevensteen, *Documents*, I, 22, 23, 24, 25, 27, 32, 33, 36, 40, 73. In Brabant’s Diest, for instance, plague processions were held in 1529, 1533 and 1564; C. De Backer, ‘Maatregelen tegen de pest te Diest in de vijftiende en zestiende eeuw’, in *De pest in de Nederlanden: medisch historische beschouwingen 650 jaar na de zwarte dood: Vierde symposium Geschiedenis der geneeskundige wetenschappen* (Brussels: Koninklijke Academie voor Geneeskunde van België, Paleis der Academiën, 1999), 183–209, at 197. Since the fifteenth century, various towns in Brabant organised plague processions, such as Leuven, Tienen, and St. Truiden; see C. Weeda, ‘Plague Policies in the Fifteenth-Century Low Countries: Duplication, Adaption and Integration’, in N. Bock and E. Lecuppre-Desjardin*, Innovation and Medieval Communities, 1200-1500* (Turnhout: Brepols, forthcoming).

77
*Chronycke van Antwerpen sedert het jaer 1500 tot 1575, gevolgd van eene beschryving van de historie ende het landt van Brabant, sedert het jaer 51 vóór tot 1565 na J.C.: volgens een onuitgegeven hs. van de 16e eeuw met fac-simile van het HS. Antwerpen*, ed. F.G. Ullens (Antwerp: Van Dieren, 1843), August 1571, 249–252.

78 Van Schevensteen, *Documents*, I, 202. Processions resumed on 28 October 1585.

79 Jean Charles Diercxsens, *Antverpia Christo nascens et crescens seu Acta ecclesiam Antverpiensem ejusque apostolos ac viros pietate conspicuos concernentia usque ad seculum XVIII*, 7 vols (Antwerp, 1773), III, 265.

80 J. Muller, *St. Jacob’s Antwerp Art and Counter Reformation in Rubens’s Parish Church* (Leiden: Brill, 2016), 312–319, 331; Thijs, *Van Geuzenstad*, 16, 20.

81 The Catholic population began to repair the Church property in 1567. Marnef, *Antwerpen in de tijd van de Reformatie*, 128, 168.

82 Muller, *St. Jacob’s Antwerp Art*, 314–315; J. Israel, *The Dutch Republic: Its Rise, Greatness, and Fall 1477–1806* (Oxford: Oxford University Press), 152.

83 Thijs, *Van Geuzenstad*, 23, 27–30.

84
*Dagboek van Jan de Pottre, 1549–1602,* ed. B. de St. Genois and C. Annoot-Braeckman (Ghent, 1861), 111–112.

85 Muller, *St. Jacob’s Antwerp Art*, 13.

86 ‘Index der gebodboeken’, 175 (1522), 265 (1559), 278 (1564), 324 (1575).

87
*Chronycke van Antwerpen*, ed. Ullens, 4 February 1572, 257–258.

88 Godevaert van Haecht, *Kroniek over de troebelen*, ed. Van Roosbroeck, 150–151.

89
*Chronycke van Antwerpen*, ed. Ullens, 4 February 1572, 257–258.

90 J. Coomans and C. Weeda, ‘Politics of Movement: Exploring Passage Points in Responses to COVID-19 and the Plague in the Fifteenth-Century Netherlands’, Journal for the History of Environment and Society, 2020, 5, 79–89.

91 Certificatieboek 1571–1572; Van Schevensteen, *Documents*, I, 101–124. 18 August 1571: ordinance of the burgomasters and aldermen of Rijssel, for inn keepers and others, that persons and goods travelling from regions where there is plague, produce a certificate, stating they come from houses or places free of infection of plague. This suggests the Antwerp authorities were responding to an external request, rather than that the issuing of certificates was integral to their own plague policy.

92 In the framework of the GIStorical Antwerp Project, Van Kooten mapped in GIS 13,331 households for which various information dated from around 1584 was gathered: the house’s name, owner and tenant’s names, owner and tenant’s occupations, etc. By cross-referencing this data with the information mentioned in the health passes, we managed to map with different levels of certainty the health passes applicant’s residences when the latter lived in Antwerp. For more details on the methodology, see Van Kooten, ‘Antwerpen 1584’, 233–5.

93 Such passes were indeed popular in Italy, especially also between 1575 and 1578. A. Bamji, ‘Health Passes, Print and Public Health in Early Modern Europe’, *Social History of Medicine*, 2017, 32/3, 441–464 at 458–459; C.M. Cipolla, *Public Health and the Medical Profession in the Renaissance* (Cambridge: Cambridge University Press, 1976), 25–26.

94 Weeda, ‘Plague Policies in the Fifteenth-Century Low Countries’, forthcoming.

95 G. Asaert, ‘De oudste certificatiën van de stad Antwerpen (1468–1482)’*, Bulletin de la Commission royale d’histoire: Académie royale de Belgique*, 1966, 132, 261–296.

96 R. Doehaerd, *Études Anversoises: Documents sur le commerce international à Anvers 1488–1514*, 3 vols (Paris: École Pratique des Hautes Études, 1962), II, 295.

97 Van Schevensteen, Documents, I, 14–15.

98
*Ibid.*, 99.

99
*Ibid*., 39–40; 43–46, 71–72, 75–76, 78, 80–81, 208–209, 210–214.

100 Asaert, ‘De oudste certificatiën’, 262.

101 Van Schevensteen, *Documents*, I, 210–213.

102 Bamji, ‘Health Passes’, 453.

103 Van Roey, ‘De sociale structuur en de godsdienstige gezindheid’, 193–194; Marnef, *Antwerpen in de tijd van de Reformatie*, 131–136.

104 165 health passes were delivered to 189 applicants of whom 66 lived in Antwerp. For 15 of those, the location of the residence was not described with enough precision to enable us to locate them, but we managed to locate 51 applicants, with different levels of precision: 43 per cent were located very precisely (the house), and 57 per cent at the level of the street.

105 Coomans, *Community, Urban Health and Environment,* 224-25.

106 Van Schevensteen, *Documents*, I, 55–71.

107
*Ibid*., 59–60. Repeated verbatim at 87.

108
*Ibid*., 59–60, 87.

109
*Ibid*., 61.

110 Cohn, *Cultures of Plague*, 103–104, 118; Naphy, *Plagues, Poisons and Potions*; Carmichael, ‘Plague Legislation’, 519; Henderson, *Florence under Siege,* 232–240.

111 Van Schevensteen, *Documents*, I, 88.

112
*Ibid*., 89.

113
*Ibid*.

114 Diercxsens, *Antverpia Christo nascens et crescens*, V, 182–183; Van Schevensteen, *Documents*, I, 79–80.

115 Van Schevensteen, *Documents*, I, 89.

116
*Ibid*., 89–91.

117
*Ibid*., 90.

118 Crawshaw, *Plague Hospitals,* 14–15; Henderson, *Florence under siege;* Carmichael, *Plague and the Poor.*

119 116 houses are mentioned in the register of which 30 lacked information about their location. We managed to map 86 houses, with different levels of precision: 45 per cent were located very precisely (on the house level), and 55 per cent on the level of the street.

120 Henderson, *Florence under Siege*, 119.

121 We managed to approximate the size of dwelling spaces by dividing for each islet an estimation of built areas (using a georeferenced version of Joan Blaeu’s map of Antwerp) by the number of registered households.

122 ‘Index der gebodboeken’, 178 (1524), 259 (1556), 261 (1557), 272 (1561), 406 (1595).

123 Of 109, we know that they were a member of the civil guard or the armed guilds and therefore we were able to retrieve information about their religious denomination: 58 (53 per cent) were Protestant, 51 (47 per cent) Catholic, which more or less fits the overall distribution of the Antwerp population.

124 We cannot reach conclusions on the absence of correlations between those variables though. The small size of our datasets (less than 100 both for locked houses and health passes applicant’s residences for 80 quarters) and the small range of values for several of our datasets (89 per cent of the districts have between 0 and 2 houses closed) complicate the picture and make conclusions difficult to be drawn. We can only see recurring similarities between the variables.

125 Carmichael, *Plague and the Poor*; G. Alfani and T. Murphy, ‘Plague and Lethal Epidemics in the Pre-Industrial World’, *Journal of Economic History*, 2017, 77, 314–343.

126 The register counts 193 deaths by plague, of which 21 were impossible to locate. We managed to map the rest (172) with different levels of precision: 44 per cent were located very precisely (the house), and 56 per cent on the level of the street.

127 Handbogengang: 5 households on 6 = 83 per cent; Paradijstraat: 4 on 57 = 7 per cent; Zwanengang: 4 on 34 = 12 per cent; Rozenstraat: 11 on 37 = 30 per cent; Apostelstraat: 4 on 19 = 21 per cent.

128 The average household size was 5.1 based on a population count in 1586. Large differences, though, existed between households correlating strongly with wealth. Rich households tended to be larger than poor households. E. Scholliers, *Loonarbeid en honger: De levensstandaard in de XVe en XVIe eeuw te Antwerpen* (Antwerp: De Sikkel N.V., 1960), 195–198.The parish records of Sint-Jacob’s document burials in that parish between 1569 and 1580, and include a separate section listing deaths from plague between 1570 and 1578. The entire register consists of 2,675 entries of parish burials between 1569 and 1580. Between 1570 and 1578, 193 plague deaths were recorded in the separate register. The years 1571, 1575 and 1577 are peak years for plague deaths. This is not reflected in the total number of deaths in the same parish, which even show a slight decrease in the number of fatalities in 1572 and after 1575, yet otherwise remain relatively stable. Caution is heeded when interpreting this data. The city also appointed special plague burial plots, such as on 17 August 1571 at the plague hospital in Nieuwstad. Also, members of the Jewish population, mostly Portuguese merchants called ‘Maranen’, who numbered several hundred residents (as well as their families), would have been buried in their own cemetery and are not included in these numbers.

129 Curtis and Roosen, ‘The Sex-Selective Impact of the Black Death’, 255–256.

130 S.K. Cohn Jr. and G. Alfani, ‘Households and Plague in Early Modern Italy’, *Journal of Interdisciplinary History*, 2007, 38/2, 177–205.

131 D.R. Curtis, ‘The Female Experience of Epidemics in the Early Modern Low Countries’, *Dutch Crossing: Journal of Low Country Studies*, 2021, 45/1, 3–20; C. Rawcliffe, *Medicine and Society in Later Medieval England* (Stroud: Alan Sutton, 1995), 182–183.

132 In Zierikzee in Zeeland in 1568 and 1573. K. Heyning, *Turbulente tijden: Zorg en materiële cultuur in Zierikzee in de zestiende eeuw* (Hilversum: Verloren, 2017), 47; Curtis, ‘The Female Experience’, 5.

133 R. Munkhoff, ‘Searchers of the Dead: Authority, Marginality, and the Interpretation of Plague in England, 1574–1665’, *Gender & History*, 1999, 11/1, 1–29.

134
*Chronycke van Antwerpen sedert het jaer 1500 tot 1575*, ed. Ullens, 249–250.

## Notes

Janna Coomans is a university lecturer with a focus on social history and public health in premodern cities. Her dissertation was published in 2021 as *Community, Urban Health and Environment in the Late Medieval Low Countries* (Cambridge University Press). She was post doc in the ERC-project ‘Healthscaping Urban Europe’, and currently works on the VENI (Dutch Research Council) project ‘Inflammable cities: how fire risks transformed the Low Countries, 1250–1600’.

Léa Hermenault is an urban historian and spatial archaeologist. She studies interactions between material, environmental constraints and social processes. She is currently post doc for CLARIAH-VL in the project ‘Belgian Historical Gazetteer’ at the University of Antwerp and lecturer at the University of Amsterdam. She was previously post doc in the ERC-project ‘Healthscaping Urban Europe’. Her dissertation was published in 2023 as *Paris entre forme et flux, circulations, échanges commerciaux et matérialité urbaine (XVe-XIXe siècle)*.

Rogier van Kooten is a socio-economic and spatial historian at the University of Antwerp. Between 2016 and 2021 he was one of the creators of ‘GIStorical Antwerp’, a four-century household-level GIS reconstruction of the city of Antwerp. He is currently studying the evolution of notarial credit networks in nineteenth-century Antwerp and surroundings (research project ‘Mapping the Market’). He also works as Education Programme Director of the N.W. Posthumus Institute (Utrecht University).

Claire Weeda is a cultural historian at Leiden University. Her research focuses on medicine, labour and power in Europe between 1000 and 1600. Recent publications include *Ethnicity in Medieval Europe: Medicine, Power and Religion* (Boydell and Brewer, 2021) and *Policing the Urban Environment in Premodern Europe*, which she co-edited with Carole Rawcliffe (Amsterdam University Press, 2019).

